# Better together: Integrating biomedical informatics and healthcare IT operations to create a learning health system during the COVID‐19 pandemic

**DOI:** 10.1002/lrh2.10309

**Published:** 2022-03-30

**Authors:** Philip R.O. Payne, Adam B. Wilcox, Peter J. Embi, Christopher A. Longhurst

**Affiliations:** ^1^ Washington University School of Medicine Institute for Informatics St. Louis Missouri USA; ^2^ Department of Biomedical Informatics Vanderbilt University Medical Center Nashville Tennessee USA; ^3^ Department of Biomedical Informatics UC San Diego Health La Jolla California USA

**Keywords:** informatics, information systems, leadership, organization and administration

## Abstract

The growing availability of multi‐scale biomedical data sources that can be used to enable research and improve healthcare delivery has brought about what can be described as a healthcare “data age.” This new era is defined by the explosive growth in bio‐molecular, clinical, and population‐level data that can be readily accessed by researchers, clinicians, and decision‐makers, and utilized for systems‐level approaches to hypothesis generation and testing as well as operational decision‐making. However, taking full advantage of these unprecedented opportunities presents an opportunity to revisit the alignment between traditionally academic biomedical informatics (BMI) and operational healthcare information technology (HIT) personnel and activities in academic health systems. While the history of the academic field of BMI includes active engagement in the delivery of operational HIT platforms, in many contemporary settings these efforts have grown distinct. Recent experiences during the COVID‐19 pandemic have demonstrated greater coordination of BMI and HIT activities that have allowed organizations to respond to pandemic‐related changes more effectively, with demonstrable and positive impact as a result. In this position paper, we discuss the challenges and opportunities associated with driving alignment between BMI and HIT, as viewed from the perspective of a learning healthcare system. In doing so, we hope to illustrate the benefits of coordination between BMI and HIT in terms of the quality, safety, and outcomes of care provided to patients and populations, demonstrating that these two groups can be “better together.”

## 1 INTRODUCTION

Biomedical research and healthcare practice is experiencing a new “data age,” where bio‐molecular, clinical, and population‐level data are increasingly available and suitable for use in support of both hypothesis generation and testing as well as operational decision‐making. Ideally, these data can and should be accessed by researchers, clinicians, and administrators to support data‐driven solutions to a broad variety of use cases, from drug discovery to point‐of‐care decision‐making to precision approaches to individual and population health.^1^, ^2^, ^3^, ^4^, ^5^, ^6^, ^7^, ^8^, ^9^, ^10^, ^11^, ^12^, ^13^ Considering these opportunities, we believe that it is important to consider the ways in which the design and delivery of biomedical informatics (BMI) and healthcare information technology (HIT) tools, methods, and expertise are coordinated and integrated. While the history of BMI includes numerous examples of situations in which both BMI and HIT researchers and practitioners partnered to deliver innovative HIT platforms at the point of care, in more recent experience, there has been a divergence between BMI and HIT, with BMI seen primarily as an academic pursuit, and HIT seen primarily as a service activity.^14^, ^15^ However, during the challenges of the COVID‐19 pandemic, there have been multiple reports demonstrating a reversal of this trend, in which BMI and HIT units have closely partnered to rapidly deliver and scale essential solutions to data capture, management, analysis, health care delivery, and ultimately, knowledge generation and dissemination, in response to an unparalleled public health emergency.^16^, ^17^, ^18^, ^19^ While these partnerships may have initially formed from an emergent need for agility, the persistence of the partnerships throughout the pandemic have improved the direct application data while also sustaining data management. *These examples during the COVID‐19 pandemic provide evidence that BMI and HIT can be synergistic disciplines that may work better together than apart to positively impact health and healthcare*.

When BMI and HIT innovation and practice are approached in an integrated manner, they can create the opportunity to build and operate a healthcare system that is able to generate data, information, and knowledge every time we interact with patients, thus improving the care that they, their families, and their communities receive.^20^, ^21^, ^22^, ^23^ Such a *learning health system* facilitates intelligent and data‐driven intervention at the individual and population levels that are able to improve the value, quality, safety, and outcomes of care, all of which are desirable outcomes. Further, by bringing BMI innovations into the clinical practice setting in a scalable and sustainable manner, as is made possible through deep collaboration with HIT practitioners, we move the EHR beyond serving as a tool that addresses medicolegal documentation and billing requirements and positions it as a platform that adds value and facilitates the best work and engagement of all stakeholders participating in the healthcare system.^24^, ^25^, ^26^

It has been our experience that improved or renewed integration and collaboration between BMI and HIT leaders, research and innovation programs, and operations, will require these respective communities of practice to adopt or renew a commitment to three critical axioms, as introduced below and explored further in the remainder of this report:

### 1.1 Axiom 1: we must conceptualize data as a renewable resource and focus on enabling responsible access to it, rather than preventing or otherwise limiting its use

Healthcare data need to be understood as having value that grows with each use and reuse, like a renewable resource. Current healthcare data management practices often focus on controlling and otherwise limiting access. This focus is motivated by concerns over data ownership, privacy, security, and the potential or intrinsic value of such data.^27^, ^28^ This contrasts with the vision of building a learning healthcare system, where the value of data increases with utilization due to improved understanding. Therefore, our focus can and should be on enabling appropriate and responsible use of such data that can generate actionable insights that serve the common good.^23^, ^29^ Achieving this vision requires the creation and management of appropriate data repositories and sharing platforms, as well as the provisioning functionality to ensure that privacy and security are maintained while simultaneously facilitating timely and ready access to such data assets. Doing so requires more than technology, but also requires novel tools, incentives, and policy frameworks that can be created through rapid‐cycle innovation spanning BMI and HIT teams.

### 1.2 Axiom 2: we must make the translation of multi‐scale data into actionable knowledge agile, timely, and expected outcome when such data are being collected and managed

In addition to considering data as a renewable resource, we must also make sense of such data and translate resulting insights into actionable knowledge. This translation should be a common and cross‐disciplinary occurrence that is acted upon rapidly and efficiently, rather than occurring only in research projects or operational “silos” (which is often the case today), often taking place with lengthy time scales. Achieving such a vision requires data analytics and knowledge management techniques that BMI investigators often develop and demonstrate via their research programs, along with the workflow‐integrated delivery systems for which HIT leaders have unique expertise and for which they are held accountable.^6^, ^14^, ^30^ Unfortunately, such activities and capabilities are commonly approached in a partitioned manner, with many BMI methods and tools being isolated to laboratory‐based settings, and never translated into the clinic, while simultaneously, HIT professionals are not necessarily engaging BMI researchers to identify and source such capabilities for use in a “production” environment. Such “informatics translation” must become the norm, and even better, be appropriately resourced and incentivized so as to make these types of efforts advantageous to both communities of practice to engage in.

### 1.3 Axiom 3: we must establish and sustain processes for the rapid return of emergent knowledge to clinicians, patients, and communities

It has regularly been shown that the use of BMI methods allows for the design of data‐driven interventions that support improved decision‐making by providers and patients, delivered in a timely and efficient manner, which can improve individual‐ or population‐level health.^6^, ^15^, ^30^, ^31^ However, implementing such interventions beyond a research setting, as noted above, often involves the use of clinical decision support, guideline delivery systems, population health management platforms, and tailored patient‐ or population‐level messaging at scale, all of which relies upon the competencies of HIT leaders and their teams. We believe that the BMI and HIT communities must return the knowledge generated via the methods and technologies they are responsible for, back to clinicians, patients, and communities, all in service of improving the human condition. Satisfying such an imperative requires the efficient and widespread use of BMI and HIT in a coordinated manner, again arguing for an integrated approach to these two areas.

## 2 EVIDENCE FOR THE ARGUMENT

### 2.1 The state of knowledge prior to the COVID‐19 pandemic

While it may be conventional wisdom that the ideas and arguments introduced above are well known and have been extensively published in the relevant literature, our assessment of the current knowledge base, particularly as it existed in the 5 years prior to the onset of the COVID‐19 pandemic, indicates otherwise, showing a relatively low level of discussion of these issues beyond the immediate informatics and public health science domains. This limits their impact on critical thinking and decision‐making beyond those primarily research‐oriented communities of practice.

**FIGURE 1:**
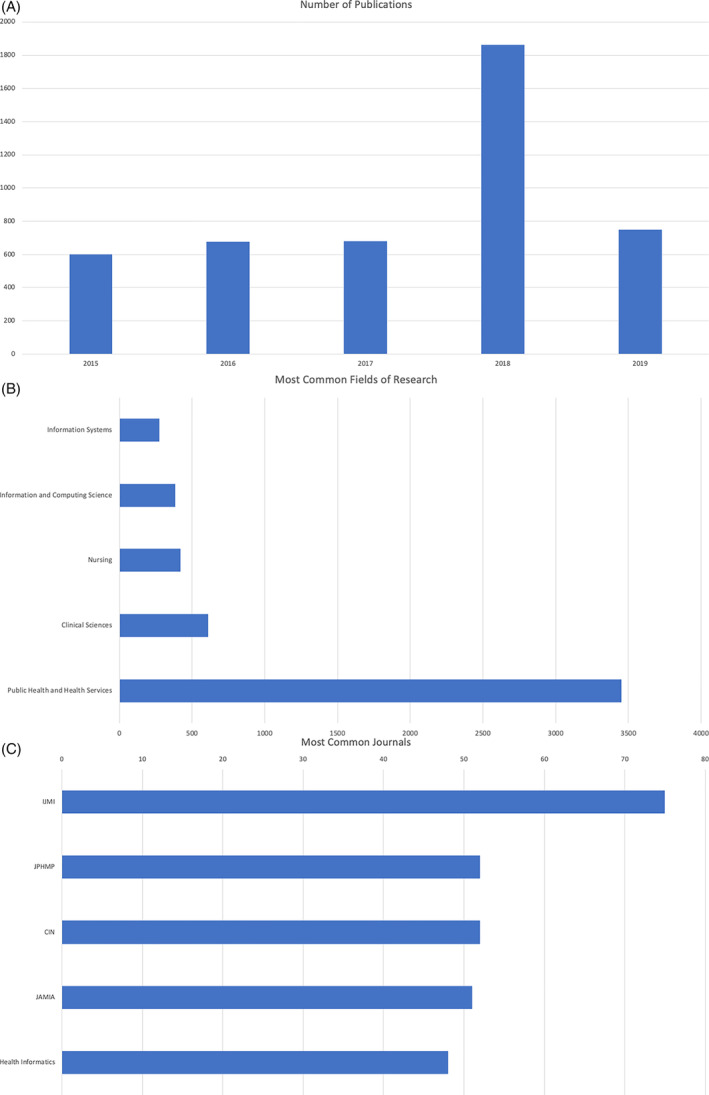
(A) Aggregate number of publications per year, indexed using the keywords “Informatics” AND/OR “Information Technology” AND “Leadership.” (B) Distribution of the publications shown in Figure 1A by domain. (C) Top five journals where the publications shown in Figure 1A are published, and the number of publications present in each such venue

Specifically, we conducted a search of the relevant literature using Google Scholar (https://scholar.google.com/) and analyzed ensuing search results using the Dimensions.AI platform (https://app.dimensions.ai/). This search was performed using the keywords “Informatics” AND/OR “Information Technology” AND “Leadership,” limited to the 5‐year prior to the onset of the COVID‐19 pandemic (2015‐2019), in the domains of “Medical and Health Sciences” OR “Public Health and Health Services,” resulting in 4566 publications. Figure 1A shows the number of articles published related to the targeted domains, stratified by year over a 5‐year period (with an average of 740 publications being indexed per year). Figure 1B shows the relative distribution of these articles by publication domain, while Figure 1C shows the top five journals in which such manuscripts or reports appear. When these results are considered as a whole, several conclusions can be drawn, as follows:

- There is a steady rate of publications in this domain (between 600 and 800 per year), but there has not been substantial growth in this rate over the 5 years shown in Figure 1, with the exception of a “surge” in such publications in 2018 (which is associated with a set of publications relevant to the fields of tobacco research as found in the public health literature);
- Many of these publications have been in domain‐specific journal relevant to the “Information Systems' and “Public Health and Health Services” communities, while a very small proportion have been published in venues targeting the broad clinical sciences or health policy communities; and
- Four of the top five journals in which such work has been published specifically target the biomedical informatics community‐of‐practice (IJMI, CIN, JAMIA and Health Informatics)

The steady rate of publications is more notable when considering that it happened during and immediately after widespread growth in EHR adoption, and as these issues were becoming more important.

### 2.2 Emerging trends in the state of knowledge with the onset of the COVID‐19 pandemic

As was introduced previously, the experiences of the past 2 years, as a function of the COVID‐19 pandemic, have shown a renewed convergence of BMI and HIT in some institutions and settings, with demonstrable benefits in terms of the ability to develop, deploy, and scale data‐centric solutions in support of our responses to the clinical and public health challenges associated with this public health emergency. Numerous reports concerning these experiences have appeared in the informatics literature in this time period, and while not meant to be an exhaustive inventory of such publications, Table 1 below provides a brief synopsis of the types of efforts and outcomes that have been described in this context. These include specific examples from the authors' own institutions, as well as reflections across multiple organizations.

**TABLE 1:** Example publications concerning the alignment of BMI and HIT to support and enable COVID‐19 response efforts

Publication	Summary of efforts and outcomes
Reeves, J. Jeffery, Hannah M. Hollandsworth, Francesca J. Torriani, Randy Taplitz, Shira Abeles, Ming Tai‐Seale, Marlene Millen, Brian J. Clay, and Christopher A. Longhurst. 2020. “**Rapid Response to COVID‐19: Health Informatics Support for Outbreak Management in an Academic Health System**.” *Journal of the American Medical Informatics Association: JAMIA* 27 (6): 853‐59.	This report described rapid‐cycle innovation projects involving the design, implementation, and evaluation of screening processes, laboratory testing protocols, clinical decision support rooks, reporting tools, and patient‐facing technologies as part of a comprehensive COVID‐19 response at UC San Diego Health. Such efforts involve close coordination of BMI and HIT leaders and practitioners, and positioned the health system to respond rapidly and efficiently to the dynamic challenges surrounding COVID‐19 diagnosis and management, as well as public health policies and interventions.^18^
Kannampallil, Thomas G., Randi E. Foraker, Albert M. Lai, Keith F. Woeltje, and Philip R. O. Payne. 2020. “**When Past Is Not a Prologue: Adapting Informatics Practice during a Pandemic**.” *Journal of the American Medical Informatics Association: JAMIA* 27 (7): 1142‐46.	This perspective introduces a pragmatic framework used at Washington University in St. Louis and BJC Healthcare in order to address functional needs in the areas of improved COVID‐19 diagnostic processes, the development of predictive models of disease spread and patient trajectories once admitted to the hospital, and the management of personnel and equipment in response to epidemiological trends. Central to this framework is the rapid “translation” of novel solutions from BMI innovation programs into scalable, enterprise‐wide HIT solutions.^19^
Dixon, Brian E., Shaun J. Grannis, Connor McAndrews, Andrea A. Broyles, Waldo Mikels‐Carrasco, Ashley Wiensch, Jennifer L. Williams, Umberto Tachinardi, and Peter J. Embi. 2021. “**Leveraging Data Visualization and a Statewide Health Information Exchange to Support COVID‐19 Surveillance and Response: Application of Public Health Informatics**.” *Journal of the American Medical Informatics Association: JAMIA* 28 (7): 1363‐73.	This report presents lessons learned from efforts spanning the state of Indiana to develop and implement population‐level dashboards that collated information on individuals tested for and infected with COVID‐19, working in partnership with state and local public health agencies as well as health systems. These efforts are situated within the context of a broader, statewide HIE, while also leveraging BMI expertise and developments produced by the teams at Indiana University and the Regenstrief Institute.^17^
Madhavan, Subha, Lisa Bastarache, Jeffrey S. Brown, Atul J. Butte, David A. Dorr, Peter J. Embi, Charles P. Friedman, et al. 2021. “**Use of Electronic Health Records to Support a Public Health Response to the COVID‐19 Pandemic in the United States: A Perspective from 15 Academic Medical Centers**.” *Journal of the American Medical Informatics Association: JAMIA* 28 (2): 393‐401.	This report presents the findings of an environmental scan of EHR‐based data‐sharing efforts at 15 academic health centers, where such data are being transmitted and analyzed in support of both public health surveillance at a regional or national level, as well as local decision‐making and operational planning. The primary outcome of the survey is a set of conclusions concerning the deleterious impacts of uncoordinated efforts at the national and regional levels, particularly as they relate to integration across and between relevant BMI and HIT knowledge and practices, that resulted in unnecessary delays in understanding, predicting, preparing for, containing, and mitigating the COVID‐19 pandemic in the US.^16^
Patel PD, Cobb J, Wright D, Turer RW, Jordan T, Humphrey A, Kepner AL, Smith G, Rosenbloom ST. “**Rapid development of telehealth capabilities within pediatric patient portal infrastructure for COVID‐19 care: barriers, solutions, results**.” *Journal of the American Medical Informatics Association: JAMIA* 2020 Jul 1;27(7):1116‐1120.	This report presents the experience from Vanderbilt University Medical Center, where operational health IT and academic informatics colleagues worked quickly at the start of the COVID‐19 pandemic to address unprecedented, surging demand for telehealth expansion in the relatively complex pediatric healthcare environment. The multidisciplinary team's design and implementation process was accomplished in a matter of days. The report describes a pathway for efficiently and robustly increasing capacity (eg, weekly telehealth visits increased 200‐fold for children aged 0‐12 years and 90‐fold for adolescents aged 13‐17 years) of remote pediatric enrollment for telehealth, while fulfilling privacy, security, and convenience concerns.^32^

From these examples, several additional conclusions can be drawn, including:

- When presented with emergent and potential existential challenges as a function of the COVID‐19 pandemic, healthcare provider organizations were able to successfully translate BMI innovations into operational tools and platforms in short time frames, with demonstrable positive impact in terms of “filling” critical data, information, and knowledge gaps.
- Common among these successes in informatics “translation” were institutions that had both academic BMI units and operational HIT units that exhibited preexisting pathways for communication and collaboration, and in many cases, had shared leadership positions that spanned such units. In each case, this was seen as critical.

When viewed collectively, we believe that these conclusions drawn from these examples, situated in the “crucible” of the COVID‐19 pandemic, are illustrative of the fundamental arguments set forth at the beginning of this report.

## 3 SUGGESTIONS FOR THE FUTURE

**FIGURE 2:**
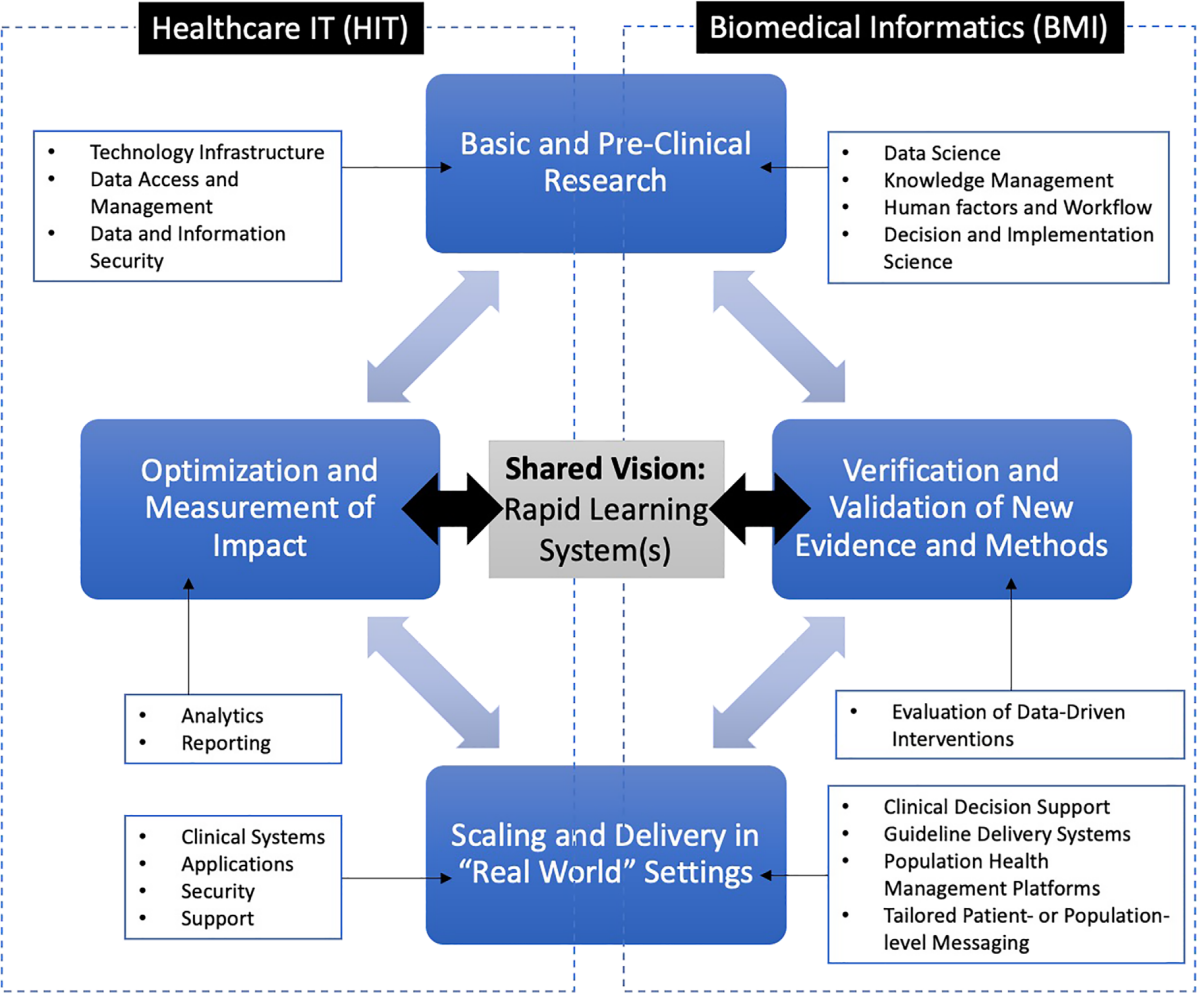
Conceptual model for a “rapid learning” healthcare system in which BMI and HIT work synergistically. In this example, four major stages for the design and implementation of a data‐driven intervention strategy are shown, spanning the capabilities of BMI and HIT leaders and practitioners. For each stage, examples of the types of competencies and methods that contribute to each such phase are shown

From our experiences, BMI and HIT can be synergistic and complementary in the modern and data‐intensive biomedical research and healthcare delivery settings. Together, they can improve the value, quality, safety, and outcomes of care. By working together, they can add to our knowledge of human disease and the ways in which we use that data, information, and knowledge to improve healthcare delivery and outcomes. However, achieving this vision beyond these examples and beyond this pandemic requires a common understanding of how these two fields can and should work together, ultimately creating what has been labeled as a “rapid learning” healthcare system (Figure 2). Such common understanding likely extends well beyond the immediate BMI and HIT communities of practice, arguing for efforts to raise this issue and its merits in the broader clinical and health policy leadership settings.

In the end, achieving such a common understanding of the synergistic natures of BMI and HIT depends on a clear recognition of why this type of collaboration is necessary and appropriate, shared respect across disciplines, and the positioning of senior leadership within organizations that have requisite BMI and HIT knowledge to ensure that they can appreciate and support such visionary and strategic approaches to capitalizing on the health “data age.” The recent experiences of the COVID‐19 pandemic have served to amplify these needs in important ways, showing that when healthcare provider organizations are placed under extreme pressure, this type of collaborative model can become central to shared success and ensuing positive impact on the health of individuals and populations.

Through realigning our collective organizational structures, incentives, funding models, and professional identities, this vision for a BMI and HIT enabled “rapid learning” health system can be realized. It is our hope that this report can serve as the catalyst for a community‐wide dialogue that can define and implement such transformative and critical changes in BMI and HIT leadership, thinking, and practice.

## CONFLICT OF INTEREST

The authors have no competing interests to declare.

## AUTHOR CONTRIBUTIONS

The authors (Philip R.O. Payne, Adam B. Wilcox, Peter J. Embi, and Christopher A. Longhurst) contributed equally to the conception of this manuscript—the acquisition, analysis, or interpretation of relevant data in support of the work—preparation of the manuscript content, and final approval of the version to be published.
